# Um Subtipo Raro de um Tumor Raro

**DOI:** 10.36660/abc.20220486

**Published:** 2023-02-13

**Authors:** Paulo Medeiros, Ana Rita Coelho, João Magalhães, Nuno Salomé, Vítor Pereira

**Affiliations:** 1 Departamento de Cardiologia Hospital de Braga Braga Portugal Departamento de Cardiologia, Hospital de Braga, Braga – Portugal; 2 Departamento de Patologia Centro Hospitalar e Universitário de São João Porto Portugal Departamento de Patologia, Centro Hospitalar e Universitário de São João, Porto – Portugal; 3 Universidade do Minho Braga Portugal Universidade do Minho, Braga – Portugal

**Keywords:** Neoplasias Cardíacas/cirurgia, Valva Mitral/cirurgia, Diagnóstico por Imagem/métodos, Imagem Multimodal/métodos, Insuficiência Cardíaca/complicações, Leiossarcoma/cirurgia

**Figura 1:**
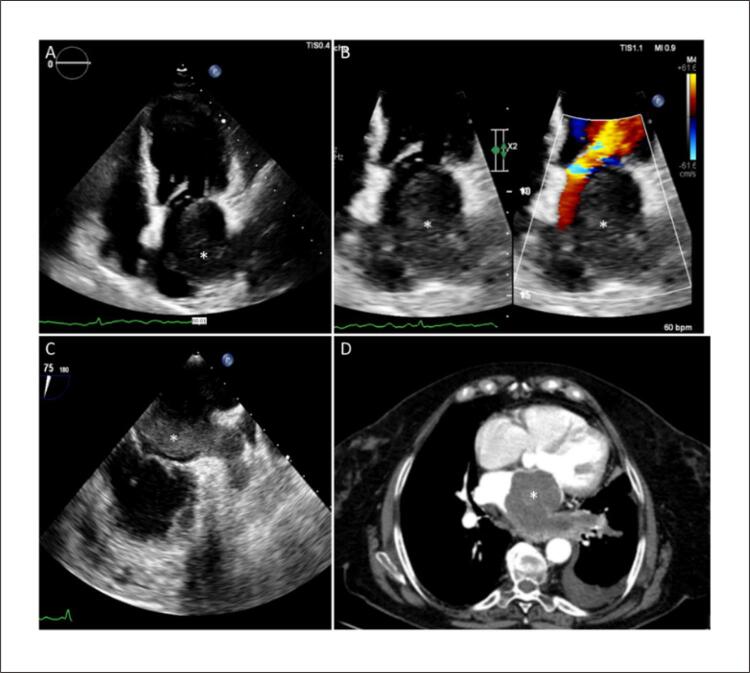
– Ecocardiografia e tomografia computadorizada. (A) Vista de quatro câmaras da ETT revelando massa oval (*) no interior do AE. (B) zoom e comparação de cores do AE e da via de entrada do ventrículo esquerdo demonstrando gradiente de pressão diastólica. (C) ETE levantando a suspeita de envolvimento da veia pulmonar superior esquerda e do apêndice atrial esquerdo. (D) TC torácica confirmando invasão das veias pulmonares pela massa.

**Figura 2:**
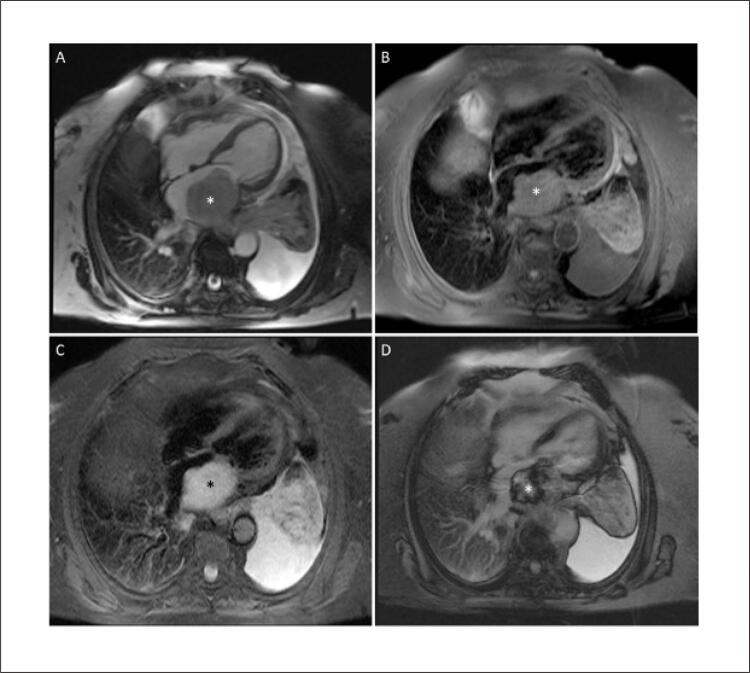
– Imagens por ressonância magnética cardíaca. (A) Imagem ponderada em T1 revelando uma massa isointensa (*). (B) Imagem ponderada em T2 com a massa aparecendo como hiperintensa. (C) Captação de contraste em sequências de primeira passagem. (D) Realce tardio pelo gadolínio heterogêneo.

Uma mulher de 71 anos apresentou fadiga, dispneia e anorexia. O exame físico revelou um sopro diastólico grave grau III no foco mitral. O ecocardiograma transtorácico (ETT) mostrou uma massa oval no interior do átrio esquerdo (AE) estendendo-se de seu teto até a válvula mitral ( Figura 1A ), causando importante gradiente de pressão transmitral ( Figura 1B ). O ecocardiograma transesofágico (ETE) ( Figura 1C ) levantou a suspeita de comprometimento da veia pulmonar superior esquerda, o que foi confirmado pela angiografia por tomografia computadorizada (TC) ( Figura 1D ). A ressonância magnética cardíaca caracterizou a massa como isointensa em imagens ponderadas em T1 e hiperintensa em T2; havia captação do contraste nas sequências de primeira passagem e realce tardio pelo gadolínio heterogêneo ( Figura 2A-D ).

**Figura 3:**
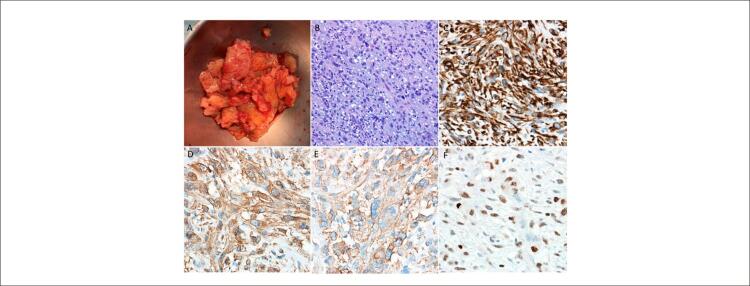
– Amostra cirúrgica e estudo histopatológico. (A) Peça cirúrgica excisada. (B) HE 200x revelando um tumor altamente celular composto por células fusiformes pleomórficas com alto índice mitótico. (C) Padrão de coloração para desmina. (D) Padrão de coloração para actina de músculo liso. (E) Padrão de coloração para h-Caldesmon. (F) Coloração nuclear para MDM2.

A paciente foi encaminhada para retirada cirúrgica da massa ( Figura 3A ). No exame histológico, determinou-se que a massa consistia em uma neoplasia maligna altamente celular com células fusiformes pleomórficas e núcleos bizarros ( Figura 3B ). A análise imuno-histoquímica revelou um padrão de coloração difuso para desmina ( Figura 3C ), actina de músculo liso ( Figura 3D ) e h-Caldesmon ( Figura 3E ). A coloração nuclear para MDM2 foi multifocal ( Figura 3F ). Em conjunto, essas características sugeriram o diagnóstico de leiomiossarcoma. Embora a cirurgia não tenha apresentado complicações imediatas, a paciente faleceu no 14º dia de pós-operatório devido a choque séptico secundário a infecção respiratória hospitalar.

Tumores cardíacos são raros e, na maioria das vezes, benignos.^1^ A apresentação clínica depende da localização, da dimensão e da mobilidade da massa. Os sarcomas representam a maioria dos tumores cardíacos malignos primários. Eles são geralmente agressivos e o prognóstico geral é ruim,^2^ com uma sobrevida mediana de cerca de 9 meses.^3^ As técnicas de imagem não invasivas têm um papel crucial na investigação diagnóstica, permitindo a localização e a caracterização tecidual da massa, bem como a avaliação do comprometimento funcional e do envolvimento das estruturas circundantes. Todas essas características contribuem para um melhor planejamento cirúrgico. No entanto, elas não diferenciam entre os subtipos histológicos.^4^ Atualmente, o único tratamento com benefício de sobrevida é a ressecção cirúrgica.^5^
