# Supplementary material for: Um Subtipo Raro de um Tumor Raro
Source: Arq Bras Cardiol. 2023 Feb 13;120(2):e20220486. [Article in Portuguese] doi: 10.36660/abc.20220486 (PMC9972779; doi:10.36660/abc.20220486)
Supplement: Translation

# Translation: A Rare Subtype of a Rare Tumor

**Authors:** Paulo Medeiros, Ana Rita Coelho, João Magalhães, Nuno Salomé, Vítor Pereira

**Affiliations:** 1 Portugal Departamento de Cardiologia, Hospital de Braga, Braga – Portugal; 2 Portugal Departamento de Patologia, Centro Hospitalar e Universitário de São João, Porto – Portugal; 3 Portugal Universidade do Minho, Braga – Portugal

**Keywords:** Heart Neoplasms/surgery, Mitral Valve/surgery, Diagnostic, Imaging/methods, Heart Failure/complications, Multimodal Imaging/methods, Leiomyosarcoma/surgery

A 71-year-old woman was admitted to our center, complaining of fatigue, dyspnea, and anorexia. Physical examination revealed a grade III low-pitched diastolic murmur at the mitral focus. Transthoracic echocardiogram (TTE) showed an oval mass inside the left atrium (LA) extending from its roof to the mitral valve ( Figure 1A ), causing significant transmitral pressure gradient ( Figure 1B ). Transesophageal echocardiogram (TEE) ( Figure 1C ) raised the suspicion of left superior pulmonary vein involvement, which was confirmed by computed tomography (CT) angiography ( Figure 1D ). Cardiac magnetic resonance imaging characterized the mass as isointense on T1 and hyperintense on T2-weightened images; there was a contrast uptake, in first-passage sequences, and a heterogeneous late gadolinium enhancement ( Figure 2A-D ).

**Figure 1:** – Echocardiography and computed tomography. (A) TTE four-chamber view revealing an oval mass (*) inside the LA. (B) zoom and color comparison of the LA and left ventricle inflow tract, demonstrating a diastolic pressure gradient. (C) TEE, raising the suspicion of left superior pulmonary vein and left atrial appendage involvement. (D) Thoracic CT scan confirming pulmonary vein invasion by the mass.

**Figure 2:** – Cardiac magnetic resonance imaging. (A) T1-weighted image revealing an isointense mass (*). (B) T2-weighted image with the mass appearing as hyperintense. (C) Contrast uptake in first-passage sequences. (D) Heterogeneous late gadolinium enhancement.

The patient was referred for the surgical removal of the mass ( Figure 3A ). Upon histologic examination, the mass consisted of a highly cellular malignant neoplasm with pleomorphic spindle cells and bizarre nuclei ( Figure 3B ). Immunohistochemical analysis revealed a diffuse staining pattern for desmin ( Figure 3C ), smooth muscle actin ( Figure 3D ), and h-Caldesmon ( Figure 3E ). Nuclear staining for MDM2 was multifocal ( Figure 3F ). Globally, these features suggested the diagnosis of leiomyosarcoma. Although the surgery had no immediate complications, the patient died on the 14^th^post-operative day due to septic shock secondary to a nosocomial respiratory tract infection.

**Figure 3:** – Surgical specimen and histopathological study. (A) Excised surgical specimen. (B) HE 200x revealing a highly cellular tumor composed of pleomorphic spindle cells with a high mitotic index. (C) Staining pattern for desmin. (D) Staining pattern for smooth muscle actin. (E) Staining pattern for h-Caldesmon. (F) Nuclear staining for MDM2.

Cardiac tumors are rare and most often benign.^1^Clinical presentation depends on the location, dimension, and mobility of the mass. Sarcomas account for most primary malignant heart tumors. These are usually aggressive and overall prognosis is poor,^2^with a median survival of about 9 months.^3^Noninvasive imaging techniques play a crucial role in the diagnostic workup, enabling location and tissue characterization of the mass, as well as an evaluation of the functional impairment and involvement of surrounding structures. All of these features contribute to a better surgical planning. However, these do not differentiate between histological subtypes.^4^Currently, the only treatment with survival benefit is surgical resection.^5^
